# Salinity Drives Functional and Taxonomic Diversities in Global Water Metagenomes

**DOI:** 10.3389/fmicb.2021.719725

**Published:** 2021-11-01

**Authors:** Huaihai Chen, Kayan Ma, Yu Huang, Yuchun Yang, Zilong Ma, Chengjin Chu

**Affiliations:** ^1^State Key Laboratory of Biocontrol, School of Ecology, Sun Yat-sen University, Shenzhen, China; ^2^State Key Laboratory of Biocontrol, School of Life Sciences, School of Ecology, Sun Yat-sen University, Guangzhou, China

**Keywords:** metagenomics, microbes, taxonomy, function, salinity, freshwater

## Abstract

A tight association between microbial function and taxonomy is the basis of functional prediction based on taxonomy, but such associations have been controversial in water biomes largely due to the probable prevalence of functional redundancy. However, previous studies on this topic used a relatively coarse resolution of ecosystem functioning, potentially inflating the estimated functional redundancy. Thus, a comprehensive evaluation of the association between high-resolution functional traits and taxonomic diversity obtained from fresh and saline water metagenomic data is urgently needed. Here, we examined 938 functionally and taxonomically annotated water metagenomes obtained worldwide to scrutinize the connection between function and taxonomy, and to identify the key driver of water metagenomes function or taxonomic composition at a global scale. We found that pairwise similarity of function was significantly associated with taxonomy, though taxonomy had higher global dissimilarity than function. Classification into six water biomes resulted in greater variation in taxonomic compositions than functional profiles, as the key regulating factor was salinity. Fresh water microbes harbored distinct functional and taxonomic structures from microbes in saline water biomes, despite that taxonomy was more susceptible to gradient of geography and climate than function. In summary, our results find a significant relationship between taxonomic diversity and microbial functioning in global water metagenomes, although microbial taxonomic compositions vary to a larger extent than functional profiles in aquatic ecosystems, suggesting the possibility and necessity for functional prediction of microorganisms based on taxonomy in global aquatic ecosystems.

## Introduction

Microbial communities are major regulators of biogeochemical processes and ecosystem functions (Hall et al., 2018). While global decline in biodiversity will negatively impact ecosystem functions and services in both aquatic and terrestrial ecosystems (Jousset et al., 2016; Schmidt et al., 2017), understanding the relationship between microbial functional profile and taxonomic composition is essential for predicting ecosystem functioning based on microbial diversity under various environmental disturbances (Torsvik and Øvreås, 2002; Wellington et al., 2003; McGill et al., 2006). It is often presumed that though microbial communities are sensitive to disturbance, their overall ecosystem functioning remains relatively stable, as many microbes are probably functionally redundant (Allison and Martiny, 2008). The association between microbial diversity and ecosystem functioning may be obscure due to functional redundancy. Yet, the extent to which such functional redundancy could affect our potential to evaluate the global consequences of shifting microbial diversity on ecosystem functioning remains largely unknown.

In contrast to the theory of functional redundancy, increasing evidence has shown that species richness can be positively related to multiple aspects of ecosystem functioning, including resource usage, nutrient cycling, and biomass accumulation (Covich et al., 2004; Downing, 2005; Balvanera et al., 2006). Compared to richness, species identity in community composition may be more suitable for predicting the ecosystem functioning. By analyzing community composition, it has been found that a decreasing gradient of microbial diversity in freshwater metagenomes could impact both broad and specialized functions (Peter et al., 2011; Delgado-Baquerizo et al., 2016a). Thus, any shift in bacterial composition could cause at least a proportional depletion of microbial capability to support ecosystem functions, implying minor functional redundancy in freshwater metagenomes. However, the characterization of ecosystem functioning of these studies was generally of low resolution, mostly in terms of certain biogeochemical processes only. A comprehensive understanding of the correlation between microbial functional and taxonomic diversities will require a thorough evaluation of microbial functional composition, which has not been analyzed and thus leaving a wide gap of knowledge that needs to be addressed.

In an analysis of global marine metagenomes, decoupling of function and taxonomy has been suggested, as the taxonomy is highly variable within specific functional groups, and environmental conditions strongly influence the distribution of functional groups while only weakly affect taxonomic composition within individual functional groups (Louca et al., 2016b). However, a lower deviation in functional profiles than taxonomic composition alone, which suggests a certain extent of functional redundancy, cannot refute the dependency of microbial functional profiles on taxonomic compositions if there exists a significant association between the two. More importantly, it has been found that salinity is the major factor regulating global aquatic microbial community, even more influential than sampling types, extreme temperature, and pH (Lozupone and Knight, 2007). Since aquatic microbes are strongly affected by physical and chemical properties of aquatic ecosystems, solely focusing on marine microbiomes may underestimate the significant effects of salinity on microbial distribution and relevant function in water biomes. Thus, it is of great importance to combine both fresh and saline water biomes in quantitative analysis to determine the relationship between functional and taxonomic diversities so as to understand the impact of future global change on diversity loss and ecosystem functioning in water biomes.

The importance of multifunctionality (Hector and Bagchi, 2007), which is the assessment of multiple functions performed by different species at the same time, has been highlighted to avoid overestimating functional redundancy (Gamfeldt et al., 2008). The microbial multifunctionality index composed by multiple assays needs to be quantified to better represent functional traits corresponding to taxonomic diversity (Bastida et al., 2016; Delgado-Baquerizo et al., 2016b). In recent decades, metagenomics has been increasingly used as a promising comparative tool (Tringe et al., 2005) to assess microbial functional diversities (Fierer et al., 2012a, b, 2013), since the abundance of each gene can be specific to a particular environmental process and numerous ecosystem functions can be examined all together in one environmental sample (Allison and Martiny, 2008). To date, open-source web servers, such as Metagenomic Rapid Annotations using Subsystems Technology (MG-RAST) (Meyer et al., 2008), are publicly available for meta-analyses (Nelson et al., 2016; Ramírez-Flandes et al., 2019) based on simultaneous metagenomic annotation against functional and taxonomic databases, allowing direct comparisons between functional profiles and taxonomic compositions. Yet, a comprehensive metagenomic analysis to elucidate the extent to which microbial functional profiles respond to different scales of variations in taxonomic compositions is still lacking for global water metagenomes.

Here, we constructed a dataset of 938 water metagenomes, functionally and taxonomically annotated, acquired from peer-reviewed publications and MG-RAST database. Based on this dataset of global water metagenomes, we specifically tested the hypotheses that (1) across the globe, microbial functional profiles are associated with taxonomic compositions, though taxonomy may harbor less similarity and larger variation than function; (2) salinity is the potential key driver for significant shifts in both functional and taxonomic diversities across the globe.

## Materials and Methods

### Data Collection

Instead of directly obtaining available shotgun metagenomic data from a public server as in previous global metagenomic studies (Nelson et al., 2016; Ramírez-Flandes et al., 2019), we strategically selected water metagenomes published in peer-reviewed journals to ensure the quality and completeness of the data. Specifically, we searched peer-reviewed publications from 2006 to 2019 from the Web of Science database using the terms such as “water metagenome,” “shotgun sequencing,” and “MG-RAST.” We included studies directly deposited or analyzed water metagenomes using shotgun sequencing without amplification in the MG-RAST database, and water metagenomes publicly available in the MG-RAST database. Based on the Study ID and/or MG-RAST ID reported in the publications, we extracted a data matrix of water metagenomes from the MG-RAST database. Specifically, in the “Analysis” function of the MG-RAST server, we typically loaded both SEED Subsystems (Overbeek et al., 2013) (Function, level 3, 2, and 1) as functional profiles and RefSeq (Tatusova et al., 2013) databases (genus, family, order, class, and phylum levels) as taxonomic compositions.

The analyses were performed using default settings (maximum *e*-value cutoff was 1e^–5^, minimum identity cutoff was 60%, and minimum alignment length was 50) (Meyer et al., 2008). We further merged the data matrix of each function extracted from different studies together to build up new datasets of microbial functional profiles annotated in the Subsystems database and taxonomic compositions annotated in RefSeq database. We chose Subsystems database for functional grouping rather than KEGG Orthology (KO; Kanehisa et al., 2015), Clusters of Orthologous Groups (COG; Galperin et al., 2014), and Non-supervised Orthologous Groups (NOG; Huerta-Cepas et al., 2015) databases because Subsystems showed diverse classification at level 1, allowing us to conduct more detailed comparison of functions among water biomes. We chose RefSeq database rather than traditional ribosomal RNA databases, such as Ribosomal Database Project (RDP; Cole et al., 2008), Greengenes (DeSantis et al., 2006), or Silva LSU/SSU (Pruesse et al., 2007) databases because taxonomic hits in RefSeq database were over 1000-fold higher than rRNA databases, which was comparable to functional hits for comparison. To expand our datasets, water metagenomes with/without assembly were both included.

**FIGURE 1:**
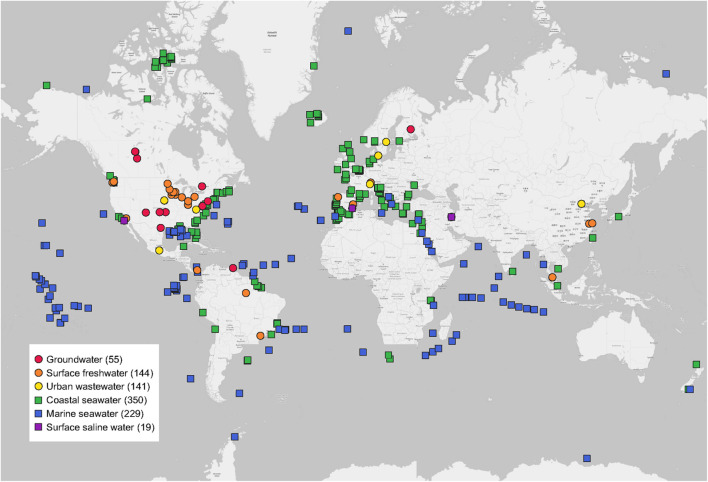
Global distribution of water metagenomes. Locations are grouped into six water biomes from 55 publications used in this study. Sample sizes of each are given in parentheses.

In total, this study included 938 water metagenomes around the world extracted from 55 MG-RAST studies published in 55 peer-reviewed papers (Figure 1). Detailed information of each water metagenome extracted from publications and the MG-RAST database was given (Supplementary Table 1). Study ID, MG-RAST ID, sample name, base pair (bp), and reads were obtained from the metadata of each water metagenome in the MG-RAST database. The geographic coordinates of latitudes (LAT) and longitudes (LONG) of each water metagenome were directly obtained from publications or MG-RAST studies. All water metagenomes were grouped into six categories, including three freshwater biomes (groundwater, surface freshwater, and urban wastewater), and three saline water biomes (coastal seawater, marine seawater, and surface saline water).

### Statistical Analyses

Hits of each functional or taxonomic category in the data matrix were standardized to relative abundance through dividing by total hits to remove bias in difference in sequencing depths and read lengths among different studies. To calculate the pairwise similarity of taxonomy, based on the relative taxonomic abundance at the genus level, we calculated the Bray-Curtis similarity following log transformation of the compositional taxonomic data by constructing a pairwise Bray-Curtis similarity matrix between each pair of two samples, which were further transformed to lists of pairwise Bray-Curtis similarities ordered by sample pair names in PRIMER 7 (Plymouth Routines in Multivariate Ecological Research Statistical Software, v7.0.13, PRIMER-E Ltd., United Kingdom). To calculate the pairwise similarity of function, based on the functional abundance at the function gene level, we calculated the Bray-Curtis similarity following log transformation of the compositional functional data by constructing pairwise Bray-Curtis similarity matrix between each pair of two samples, which were further transformed to lists of pairwise Bray-Curtis similarities ordered by sample pair names in PRIMER 7. To examine the relationship between functional and taxonomic diversities, Pearson’s correlations were constructed between the transformed lists of pairwise Bray-Curtis similarity of metagenomes annotated using the Subsystems database at Level 3 (Function) and the RefSeq database at the genus level (Taxonomy). These data processing approaches for the analyses followed the requirement of processing relative abundance of compositional data (Gloor et al., 2017). The box plots were constructed based on the pairwise similarity of function and taxonomy to compare similarity ranges of functional and taxonomic compositions related to the aquatic metagenomes. The triangular pairwise Bray-Curtis similarity matrix was used to analyze functional and taxonomic composition structures of water metagenomes by principal coordinates analysis (PCoA) and permutational multivariate analysis of variance (PERMANOVA) among biomes in PRIMER 7. Linear and non-linear regression models were constructed to examine whether functional diversity depends on taxonomic diversity. In addition, the RELATE analysis was performed to evaluate the relatedness between functional and taxonomic diversities by calculating a Spearman’s Rho correlation coefficient in PRIMER 7. Difference in the relative abundance of dominant microbial functional and taxonomic compositions for fresh vs. saline and among six water biomes was analyzed by the linear discriminant analysis effect size (LEfSe) method^1^ (Segata et al., 2011). Heatmaps were constructed using HeatMapper (Babicki et al., 2016). The significance level was set at α = 0.05 unless otherwise stated.

Co-occurrence network analysis was performed using the Molecular Ecological Network Analyses Pipeline^2^ (Zhou et al., 2011; Deng et al., 2012). The data matrix of standardized relative abundance multiplied by 10^6^ that meets the requirements of the pipeline was uploaded to construct the network with default settings, including (1) only keeping the species present in more than a half of all samples; (2) only filling with 0.01 in blanks with paired valid values; (3) taking logarithm with recommended similarity matrix of Pearson’s correlation coefficient; (4) calculation order to decrease the cutoff from the top using regress Poisson distribution only. A default cutoff value (similarity threshold, *S*_*t*_) for the similarity matrix was generated to assign a link between the pair of species. Then, the global network properties, the individual nodes’ centrality, and the module separation and modularity calculations were run based on default settings using greedy modularity optimization. Network files were exported and visualized using Cytoscape software (Shannon et al., 2003).

## Results and Discussion

### Correlation Between Function and Taxonomy

**FIGURE 2:**
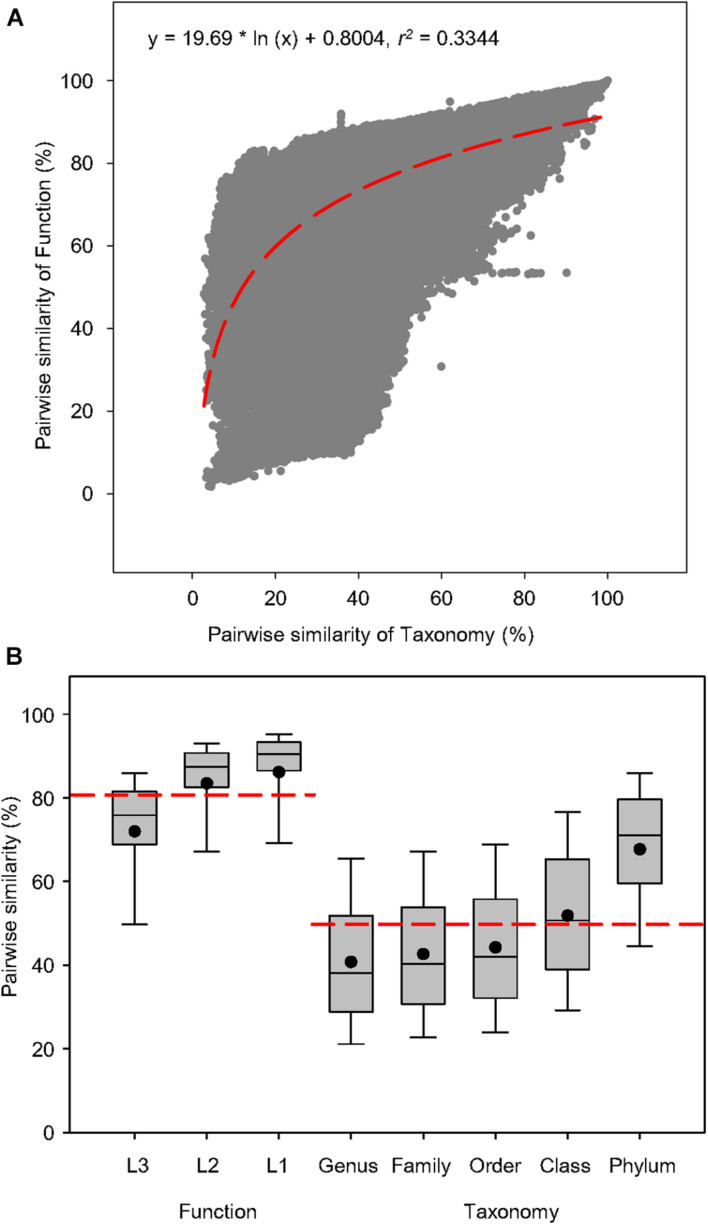
Pairwise similarity of functional and taxonomic diversities. **(A)** Logarithmic regression between pairwise Bray-Curtis similarity of water metagenomes annotated in the Subsystems database at level 3 (Function) and RefSeq database at the genus level (Taxonomy). Regression equations and coefficients are given. The logarithmic regression line was shown as the red dashed line. **(B)** Box plots and mean values of pairwise Bray-Curtis similarity of water metagenomes annotated in the Subsystems database at levels 3, 2, and 1 (Function) and RefSeq database at genus, family, order, class, and phylum levels (Taxonomy). The mean values of pairwise Bray-Curtis similarity was shown as the red dashed line.

To examine the connection between function and taxonomy, a total of 938 water metagenomes was used to create 439,453 pairwise comparisons of Bray-Curtis similarity between functional and taxonomic diversities to find out whether the correlation of function and taxonomy was significant in the global aquatic metagenomes (Figure 2). The correlations showed that pairwise similarity of function was positively correlated to that of taxonomy as indicated by the logarithmic regression (*r*^2^ = 0.3344, *P* < 0.0001) (Figure 2A), which had higher coefficient than the linear regression (*r*^2^ = 0.3209, *P* < 0.0001) (Supplementary Figure 1). This suggested that in water microbiomes, the variation of functional traits responds positively to taxonomic shifts, so the dependency of microbial function on taxonomy could be expected across fresh and saline water biomes. The box plots were constructed based on the pairwise similarity of function and taxonomy to compare similarity ranges of functional and taxonomic compositions related to the aquatic metagenomes. For the functional compositions at specific function gene levels, the average pairwise similarity of function at three levels of SEED Subsystems was greater than taxonomy at five phylogenetic levels (Figure 2B), suggesting that the variation of function and taxonomy had different sensitivity to environmental selection.

Since functional composition of higher resolution can be yielded from metagenomic analyses, we were able to better elucidate its relationship with taxonomic diversity in microbiomes than previous studies. Cross-biome soil metagenomics have shown that alpha diversity levels of microbial community were significantly associated with functional attributes (Fierer et al., 2012b). Thus, community richness can be potentially useful for inferring functional diversity. Based on regional metagenomics of soil microbial communities, a strong correlation has been observed between functional and taxonomic community composition (Fierer et al., 2013), suggesting a potential connection between beta diversities of taxonomy and function across global microbiomes. Our results further strengthened this argument by demonstrating significant associations between shifts in microbial taxonomy and functional variation in the water microbial community across the globe. This is also strong evidence for an insignificant, if any, functional redundancy in microbes at this resolution.

### Functional and Taxonomic Diversities Across Water Biomes

**FIGURE 3:**
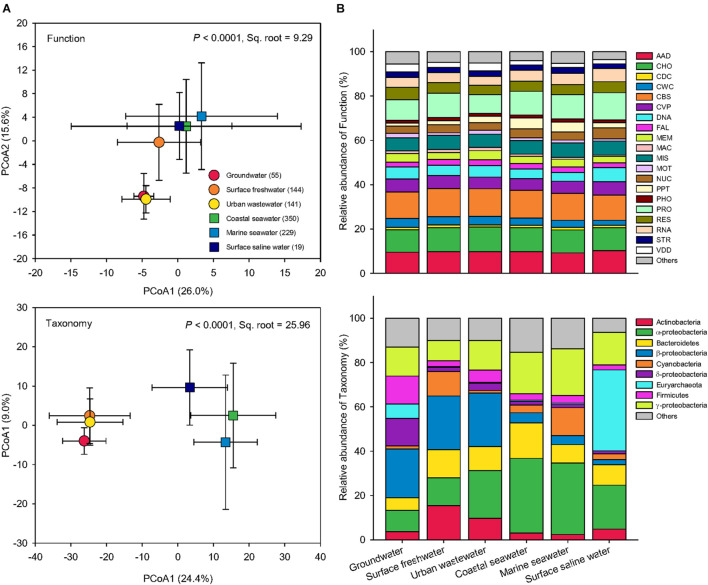
Functional and taxonomic diversities across six water biomes. **(A)** Principal coordinates analysis (PCoA) showing beta-diversity of water metagenomes annotated in the Subsystems database at level 3 (Function) and RefSeq database at the genus level (Taxonomy) among six biomes. The error bars represent the standard deviation of data ranges. Sample sizes of each biome are given in parentheses. Variations explained by two principal coordinate dimensions are given in parentheses by percentage. *P* values and Sq. root of PERMANOVA are also given. **(B)** Relative abundance of dominant water metagenomes (mean >1%) annotated in the Subsystems database at level 1 (Function), and the RefSeq database at the phylum/class levels (Taxonomy) among six water biomes. AAD, amino acids and derivatives; CHO, carbohydrates; CDC, cell division and cell cycle; CWC, cell wall and capsule; CBS, clustering-based subsystems; CVP, cofactors, vitamins, prosthetic groups, pigments; DNA, DNA metabolism; FAL, fatty acids, lipids, and isoprenoids; MEM, membrane transport; MAC, metabolism of aromatic compounds; MIS, miscellaneous; MOT, motility and chemotaxis; NUC, nucleosides and nucleotides; PPT, phages, prophages, transposable elements, plasmids; PHO, phosphorus metabolism; PRO, protein metabolism; RES, respiration; RNA, RNA metabolism; STR, stress response; VDD, virulence, disease and defense.

Based on pairwise Bray-Curtis similarity, Principal Coordinates Analysis (PCoA) was performed to examine how microbial functional profiles and taxonomic compositions differ globally among water biomes (Figure 3). The PERMANOVA showed that both function and taxonomy of water microbiomes among the six water biomes were significantly distinct (*P* < 0.0001). Compared to taxonomy (Pseudo-*F* = 70.41, Sq. root = 25.96), function had lower variation (Pseudo-*F* = 48.16, Sq. root = 9.29) across global water biomes. Greater between-sample dissimilarity in taxonomy than function suggests that the taxonomic structure of microbial communities was more sensitive to environmental selection, though environmental conditions also influence the functional traits in microbial communities. Specifically, for functional distribution, the three saline water biomes (coastal seawater, marine seawater, and surface saline water) were grouped together, whereas among the three freshwater biomes groundwater and urban wastewater were distributed near each other with surface freshwater in the middle (Figure 3A). The taxonomic compositions showed that the three freshwater biomes clustered together and were distinct from the three saline water biomes (Figure 3A), suggesting a key role of salinity in driving taxonomy and function of water microbiomes.

The relative abundances of functions were similar across global water microbiomes, while the taxonomy of microbial community was significantly more variable across the six water biomes (Figure 3B). Similar functional structures but high taxonomic variability have also been found in microbial communities from replicates of “miniature” aquatic ecosystems (Louca et al., 2016a). It is often assumed that genome streamlining (Morris et al., 2012) and horizontal gene transfer (David and Alm, 2011), common in prokaryotic populations, have contributed to similar functions performed by distinct taxa. In addition, it has been suggested that the taxonomic composition may be influenced by environmental factors distinct from those affecting the functional structure of microbial communities (Louca et al., 2018).

In functional profiles, the most significant difference was observed in genes involved in virulence, disease, and defense (VDD) as indicated by the LEfSe analysis (Figure 3B). These genes were markedly more abundant in groundwater and urban wastewater than the others, possibly because these waters were closer to human activity and hence more susceptible to pathogen and disease, leading to the proliferation of genes associated with VDD functions, such as antibiotic resistance genes (Czekalski et al., 2014). Coastal and marine seawater had greater proportion of genes associated with functions related to phages, prophages, transposable elements, and plasmids. Because the oceans are the largest reservoir of genetic diversity of virus (Suttle, 2007), the importance and focus of aquatic virology are perhaps most evident and mainly on the viromes of marine environments (Jover et al., 2014).

In terms of taxonomic compositions, surface freshwater and urban wastewater harbored the highest proportion of Actinobacteria among the biomes, while Euryarchaeota was the most prevelent in groundwater and surface saline water (Figure 3B). In addition, the two ocean biomes of coastal seawater and marine seawater were dominated by α-proteobacteria and γ-proteobacteria. The percentage of β-proteobacteria was greater in the three freshwater microbiomes than the others. Groundwater had the highest proportion of δ-proteobacteria. In agreement with our results, it has been found that α-proteobacteria and γ-proteobacteria were significantly enriched in marine ecosystems, while β-proteobacteria favored fresh environment (Hu et al., 2014; Morrissey and Franklin, 2015; Pavloudi et al., 2017). Many halotolerant bacteria with the capability to grow in a wide range of salinities are found to belong to the class of α-proteobacteria and γ-proteobacteria (Etesami and Beattie, 2018). Our extensive lines of evidence suggest that the global response of Proteobacteria was class-specific, mainly due to salinity effects. In conclusion, these striking differences in the relative abundance of dominant groups confirmed that the taxonomy of water microbiomes was more variable than function across global water biomes.

### Salinity Is the Key Regulator

**FIGURE 4:**
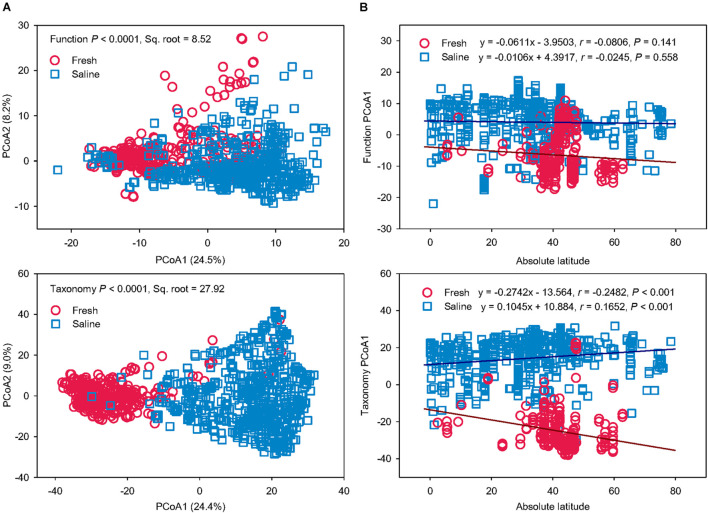
Salinity drives microbial functional and taxonomic diversities. **(A)** Principal coordinates analysis (PCoA) showing beta-diversity of microbial functional and taxonomic diversities between fresh vs. saline water metagenomes annotated in Subsystems L3 (Function) and RefSeq at the genus level (Taxonomy). The error bars represent the standard deviation of data ranges. Variations explained by two principal coordinate dimensions are given in parentheses by percentage. *P* values and Sq. root of PERMANOVA are also given. **(B)** Pearson’s correlations of the absolute latitude of water metagenome locations with principal coordinate (PCoA) 1 scores of Bray-Curtis similarity of fresh vs. saline water metagenomes annotated in Subsystems L3 (Function) and RefSeq at the genus level (Taxonomy). Regression equations, coefficients (*r*), and *P* values are given. The linear regression lines of fresh and saline water metagenomes were shown as the brown and blue solid lines, respectively.

To test if salinity is a key driver for microbial function and taxonomy, microbiomes from a freshwater environment were compared with those from saline water (Figure 4). The PERMANOVA revealed that fresh and saline water microbiomes had distinct functional and taxonomic structures (*P* < 0.0001) (Figure 4A). The differences in function (Pseudo-*F* = 112.37, Sq. root = 8.52) and taxonomy (Pseudo-*F* = 221.30, Sq. root = 27.92) between fresh vs. saline water biomes were comparable to the variation caused by grouping the microbiomes according to the six water biomes, showing that salinity can be the key factor regulating the variation of function and taxonomy in water microbiomes. However, it should be noted that determinations of salinity in this study are qualitatively based on the descriptions in the publications or MG-RAST metadata files rather than on direct measurements of salinity levels. The community variation in both function and taxonomy in saline water biomes was larger than fresh water microbiomes, probably due to much wider distribution of sampling locations of coastal and marine seawater.

To find out whether latitude gradient also drives taxonomic and functional diversities besides salinity, Pearson’s correlations were performed between the absolute latitude of sampling locations and PCoA1 scores of functional and taxonomic beta-diversity structures in fresh and saline water microbiomes, respectively (Figure 4B). Functional profiles showed no significant latitudinal gradient in either fresh or saline water microbiomes (*P* > 0.05). On the contrary, taxonomic compositions in both fresh and saline environments had significant associations with the absolute latitude (*P* < 0.001). With increasing latitude, microbial taxonomy in freshwater biomes became more different from that in the saline water biomes. Latitudinal diversity gradient, a decline of biodiversity with latitude (Hillebrand, 2004), has been found in alpha-diversity of microbes in the terrestrial (Zhou et al., 2016) and aquatic (Fuhrman et al., 2008; Huerta-Cepas et al., 2015) environments, probably attributable to temperature gradient. For the first time, we further showed this latitudinal correlation of beta-diversity in microbial taxonomic compositions rather than functional profiles in the marine ecosystems. These results suggest that global environmental drivers, such as geography and climate factors, are impacting microbial diversity in aquatic ecosystems, but the key regulator for functional traits was solely salinity in water biomes.

**FIGURE 5:**
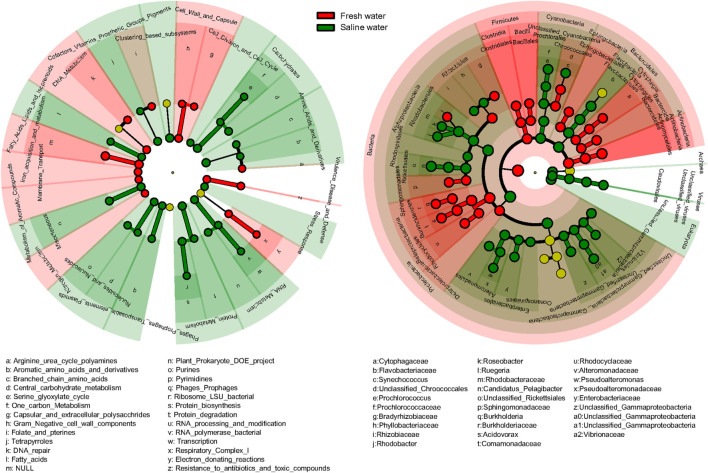
Difference of functional and taxonomic compositions between fresh and saline water. Linear discriminant analysis effect size (LEfSe) method showing the significant differences in the relative abundance of dominant microbial functional and taxonomic compositions (mean >1%) between fresh vs. saline water metagenomes annotated in Subsystems (Function) and RefSeq (Taxonomy). From the center outward, in the left panel, each circle represents level 1, 2, and 3 of functional annotation, and in the right panel, each circle represents the level of domain, phylum, class, order, family, and genus from taxonomic annotation, respectively. The functional and taxonomic compositions with significant differences are labeled by colors.

Significant differences in function and taxonomy between fresh and saline water microbiomes were revealed by LEfSe analysis (Figure 5). Generally, functional profiles of freshwater microbiomes were dominated by metabolisms of aromatic compounds, iron, and nitrogen, membrane transport and cell wall and capsule, and virulence, disease, and defense, focusing more on functions associated with nutrient cycling and defense mechanisms (Figure 5). By comparison, in the saline microbiomes’ functional profiles, functions related to primary metabolisms of nucleotides, amino acids, proteins, and carbohydrates were more abundant (Figure 5). In taxonomy, freshwater microbiomes were dominated by Actinobacteria, Clostridia, and Bacilli of Firmicutes, Burkholderiales, and Rhodocyclales belonging to β-proteobacteria and δ-proteobacteria (Figure 5). On the contrary, saline water biomes had higher abundance of Archaea, Eukaryota, and viruses. Among bacteria, only γ-proteobacteria had consistently higher abundance at class or lower levels in the saline environment, though Bacteroidetes, Cyanobacteria, and α-proteobacteria were also dominant at the phylum levels in saline water microbiomes (Figure 5). Our results suggested that microorganisms in water environments are apparently under strong selective pressures exerted by salinity, so the general biogeochemical properties, such as salinity, still primarily determine the linkage between function and taxonomy. It should be noted that the salinity is just one factor that could be considered in this study, as not all the metagenomes included had their metadata of all/certain environmental variables reported. Thus, it became really difficult to summarize and analyze a full list of environmental variables on the diversity of taxonomy and function of metagenomes, which may not rule out the effects of co-varying factors. Future work should target a complete evaluation of environmental variables to pinpoint specific environmental factors to drive both taxonomic and functional diversities in aquatic microbes.

### Functional and Taxonomic Co-occurrence Networks

**FIGURE 6:**
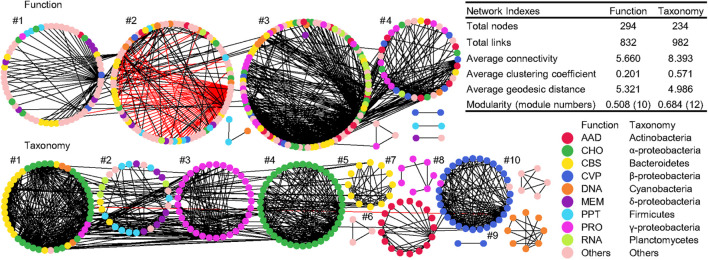
Co-occurrence networks of functional and taxonomic compositions. Network graphs with submodule structures of water metagenomes annotated in Subsystems L3 (Function) and RefSeq at the genus level (Taxonomy). Node color represents classification of Subsystems L1 (Function) or RefSeq at the phylum/class levels (Taxonomy). A black edge indicates a positive interaction between two nodes, while a red edge indicates a negative interaction. Summary of key network indexes is given in the table.

Co-occurrence networks of function and taxonomy were generated to examine the potential interaction of functional and taxonomic compositions in global water biomes (Figure 6). Network graphs with submodule structures and key network indexes indicated similar network topology and complexity between functional and taxonomic interaction patterns. The functional network had 738 positive interactions and 94 negative links while taxonomy only had positive interactions (Figure 6). Within each module, functional nodes were classified as diverse functional categories. However, submodule structures of taxonomy showed that each module was comprised of genera that were mainly from the same phylum or class, such as γ-proteobacteria in module #3 and 7, α-proteobacteria in module #4, Bacteroidetes in module #5, γ-actinobacteria in module #6, β-proteobacteria in module #8, and Cyanobacteria in module #9 (Figure 6).

## Conclusion

Our findings provide what is, to our knowledge, the first direct global metagenomic evidence to support that microbial functions are significantly correlated to taxonomy in aquatic ecosystems, mainly because salinity drives microbial functional and taxonomic diversities at the global level. However, the taxonomic diversity was more significantly associated with latitudinal gradient than the functional diversity. Our study has also brought some new insights regarding the functional redundancy of water microbiomes: while functional redundancy is probably inevitable, zooming into tens of thousands of specific functional genes like this study could significantly increase the dependency of function on taxonomy. Thus, these results have significant implications for developing microbial-ecosystem models for functional prediction based on microbial taxonomy.

## Data Availability Statement

The datasets presented in this study can be found in online repositories. The names of the repository/repositories and accession number(s) can be found in the article/Supplementary Material.

## Author Contributions

HC conceived the study, performed the data analysis, interpreted the results, and drafted the manuscript. YY, ZM, and CC secured the research funding. KM, YH, YY, and ZM critically assessed and interpreted the findings. All authors discussed results, commented on, edited, revised, and approved the manuscript.

## Conflict of Interest

The authors declare that the research was conducted in the absence of any commercial or financial relationships that could be construed as a potential conflict of interest.

## Publisher’s Note

All claims expressed in this article are solely those of the authors and do not necessarily represent those of their affiliated organizations, or those of the publisher, the editors and the reviewers. Any product that may be evaluated in this article, or claim that may be made by its manufacturer, is not guaranteed or endorsed by the publisher.

## Footnotes

1
http://huttenhower.sph.harvard.edu/lefse/

2
http://ieg4.rccc.ou.edu/MENA/
