# Supplementary material for: Salinity Drives Functional and Taxonomic Diversities in Global Water Metagenomes
Source: Front Microbiol. 2021 Nov 1;12:719725. doi: 10.3389/fmicb.2021.719725 (PMC8591284; doi:10.3389/fmicb.2021.719725)

**Supplementary Table 1. Information of MG-RAST soil metagenomes.** Data are extracted from publications used in this study including study ID, MG-RAST ID, sample name, publication, bp, reads, latitude and longitude, climate zone, climate code, mean annual temperature (MAT, °C), mean annual precipitation (MAP, mm yr-1), soil C, pH, Sand (%), Silt (%), Clay (%), and hits in Subsystems and RefSeq databases for broad and narrow functions.

Available in a separated Excel spreadsheet file.

**Supplementary figure legends**

**Supplementary Fig. 1.** Pearson’s correlations between pairwise Bray-curtis similarity of soil metagenomes annotated in Subsystems database at Level 3 (Function) and RefSeq database at genus level (Taxonomy).

**Supplementary Fig. 1.**
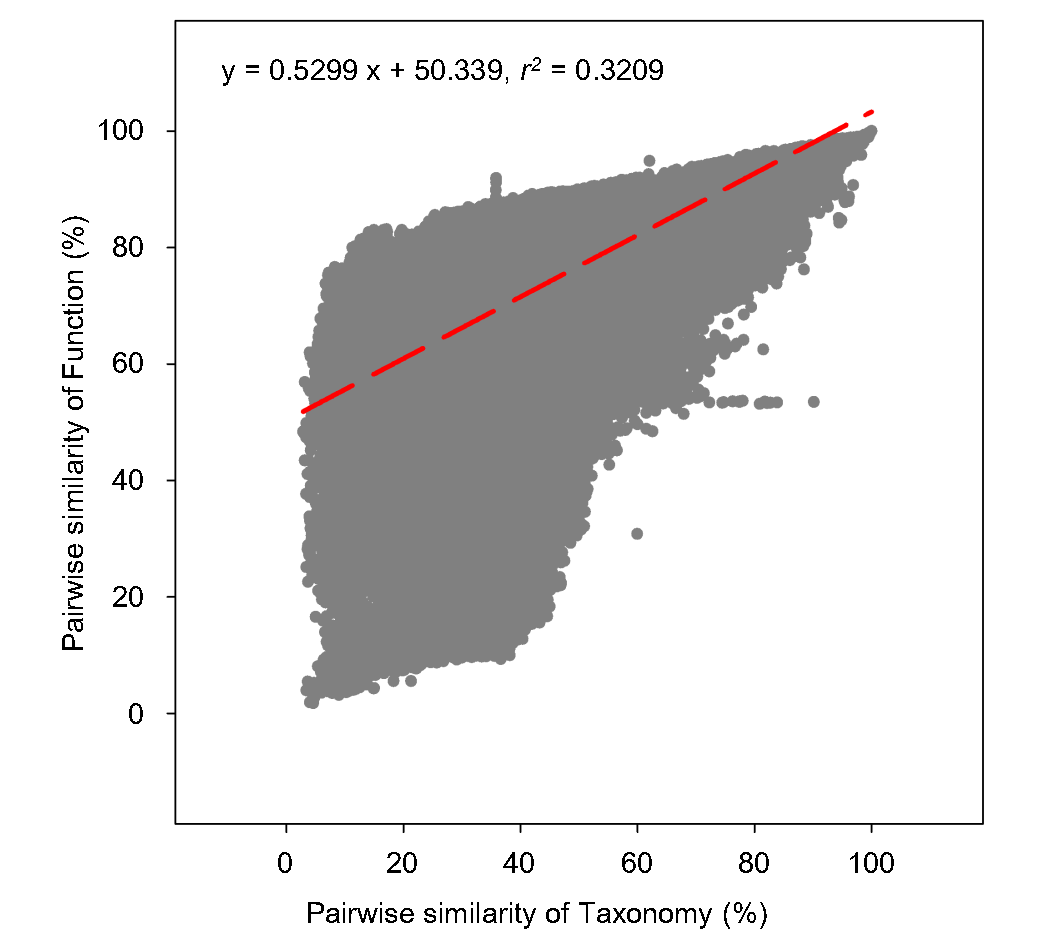

Supplement: Supplementary file 1 [file Data_Sheet_1.DOCX]
